# Automatic detection of non-perfusion areas in diabetic macular edema from fundus fluorescein angiography for decision making using deep learning

**DOI:** 10.1038/s41598-020-71622-6

**Published:** 2020-09-15

**Authors:** Kai Jin, Xiangji Pan, Kun You, Jian Wu, Zhifang Liu, Jing Cao, Lixia Lou, Yufeng Xu, Zhaoan Su, Ke Yao, Juan Ye

**Affiliations:** 1 Department of Ophthalmology, The Second Affiliated Hospital of Zhejiang University, College of Medicine, Hangzhou, 310009 China; 2 College of Computer Science and Technology, Zhejiang University, Hangzhou, 310027 China

**Keywords:** Eye diseases, Image processing, Machine learning

## Abstract

Vision loss caused by diabetic macular edema (DME) can be prevented by early detection and laser photocoagulation. As there is no comprehensive detection technique to recognize NPA, we proposed an automatic detection method of NPA on fundus fluorescein angiography (FFA) in DME. The study included 3,014 FFA images of 221 patients with DME. We use 3 convolutional neural networks (CNNs), including DenseNet, ResNet50, and VGG16, to identify non-perfusion regions (NP), microaneurysms, and leakages in FFA images. The NPA was segmented using attention U-net. To validate its performance, we applied our detection algorithm on 249 FFA images in which the NPA areas were manually delineated by 3 ophthalmologists. For DR lesion classification, area under the curve is 0.8855 for NP regions, 0.9782 for microaneurysms, and 0.9765 for leakage classifier. The average precision of NP region overlap ratio is 0.643. NP regions of DME in FFA images are identified based a new automated deep learning algorithm. This study is an in-depth study from computer-aided diagnosis to treatment, and will be the theoretical basis for the application of intelligent guided laser.

## Introduction

Diabetic retinopathy (DR) is one of the leading causes of preventable vision loss worldwide^1^. When the macula is affected in diabetic patients, it leads to diabetic macular edema (DME) which is sometimes also considered as vision-threatening and advanced stage of DR. Automated DR screening using artificial intelligence based on retinal images was not only found to be accurate, but also cost effective^2,3^. The Early Treatment of Diabetic Retinopathy Study (ETDRS) established the definition of clinically significant macular edema (CSME) for which grid/focal laser photocoagulation can significantly reduce the risk of visual acuity (VA) loss^4^. A standard set of definitions that describes the severity of retinopathy and macular edema are critical in clinical decision making. Recently, anti-vascular endothelial growth factor (VEGF) therapies were proved more effective than laser photocoagulation and other treatments^5,6^. Laser photocoagulation should be modified to be more effective.

To make an intelligent treatment decision for DME, it needs to align planned treatment locations precisely that are defined on the FFA images and actual sites on retina during the treatment. DR lesions detection using fundus images and FFA images, as non-perfusion (NP) areas and microaneurysms, is crucial for planning laser treatment locations^7^. This is a difficult job due to the fact that there is a large variation in the overall size, shape, location, and intensity of the retinal pathologies^8^. There are some studies on automated classification of retinal pathologies in fundus images for DR screening, however few studies have published an automated method to detect the lesions in FFA images^9,10^. Annotating lesions on FFA images is costly. Automatic lesion detection of retinal pathologies in FFA images will aid clinicians in both treatment and referrals.

Non-perfusion areas (NPA) of the retina are associated with the development of vascular occlusion or capillary closure. It is obvious that early detection of small isolated NPA for DR patients is crucial. NPA is one of the primary lesions occurring in DR, the grading of its severity is still based on indirect signs of ischemia detected on color fundus photographs (CFPs) in daily routine practice. A series of automatic DR lesions detection algorithms based on CFPs provided indirect information on the status of retinal capillary perfusion. Classical machine learing method was used for detection and classification of exudates and cotton wool spots in CFPs^11^. Later, a deep learning system was able to detect lesions of referable DR in CFPs ^12^. Automatic detection of NPA not only provide a fast and consistent approach but also present a cost-effective and reliable methodology compared to analysis performed by clinician observers^13^.

In this study, we identify lesions and segmentation of non-perfusion areas based on deep learning using FFA images, which is significance for DR and DME management. On the basis of the previous work, this study is an in-depth study from computer-aided diagnosis to treatment. The achievements of this study will be the theoretical basis for the application of intelligent laser model into clinical translational medicine.

## Methods

### Dataset

221 patients (435 eyes) with DME (age range 31–81 years) who visited the Eye Center at the Second Affiliated Hospital of Zhejiang University, during a period of 27 months from August 2016 to October 2019, were recruited to receive a FFA using tabletop systems HRA-II at 30° (Heidelberg, Germany), at 768 × 768 pixels. Pupil dilation was achieved with topical 1.0% tropicamide. Subjects were excluded from the study if they had a medical condition that prevented dilation or had overt media opacity. All participants signed an informed consent form prior to participation in the study. This study was approved by the Medical Ethic Committee of the Second Affiliated Hospital, Zhejiang University, and it was compliant with Declaration of Helsinki.

### Annotation of lesions

A local database of FFA images was annotated independently by 3 ophthalmologists to classify the images containing important clinical findings as microaneurysms, non-perfusion (NP) regions and leakage. Manually segmented ground-truth images were required to assess NP regions detection performance of the algorithm. A binary map for each FFA image was created based on the consensus of 3 ophthalmologists (Fig. 4). Three thousand, and fourteen images mutually agreed upon for the accuracy of their clinical label were used. Of these, 2,412 were included in the training set and 602 in the testing set, 482 were included in the validation set. The dataset contained 2,801 images of microaneurysms, 1565 images of NP regions, 579 images of leakage (Table 1).

**Table 1:** Population characteristics of FFA images.

Type	Age (years)	Male sex (%)	OD/OS	Number of images
Normal	67 ± 9	51.6	0.91	157
NP	56 ± 10	62.0	0.92	1,565
Microaneurysm	56 ± 10	60.0	0.96	2,801
Leakage	54 ± 11	59.9	0.82	579
Total	57 ± 11	60.1	0.61	3,014

### Image preprocessing

The FFA images were normalized for contrast using enhancement algorithm^14^, which is not only beneficial for further processing by computer algorithms, but also for a better evaluation of the fundus by clinicians. Contrast is then enhanced in the luminosity channel of L*a*b* color space by CLAHE (contrast limited adaptive histogram equalization). The CLAHE divides the image into small regions called tiles; the histogram on each tile is equalized so that local contrast is enhanced.

Data augmentation was then performed using horizontal and vertical filliping, rotating and adding Gaussian noise to balance the amounts of images of each classification to increase the robustness of the algorithm.

### Classification of lesions via CNN

**Figure 1:**
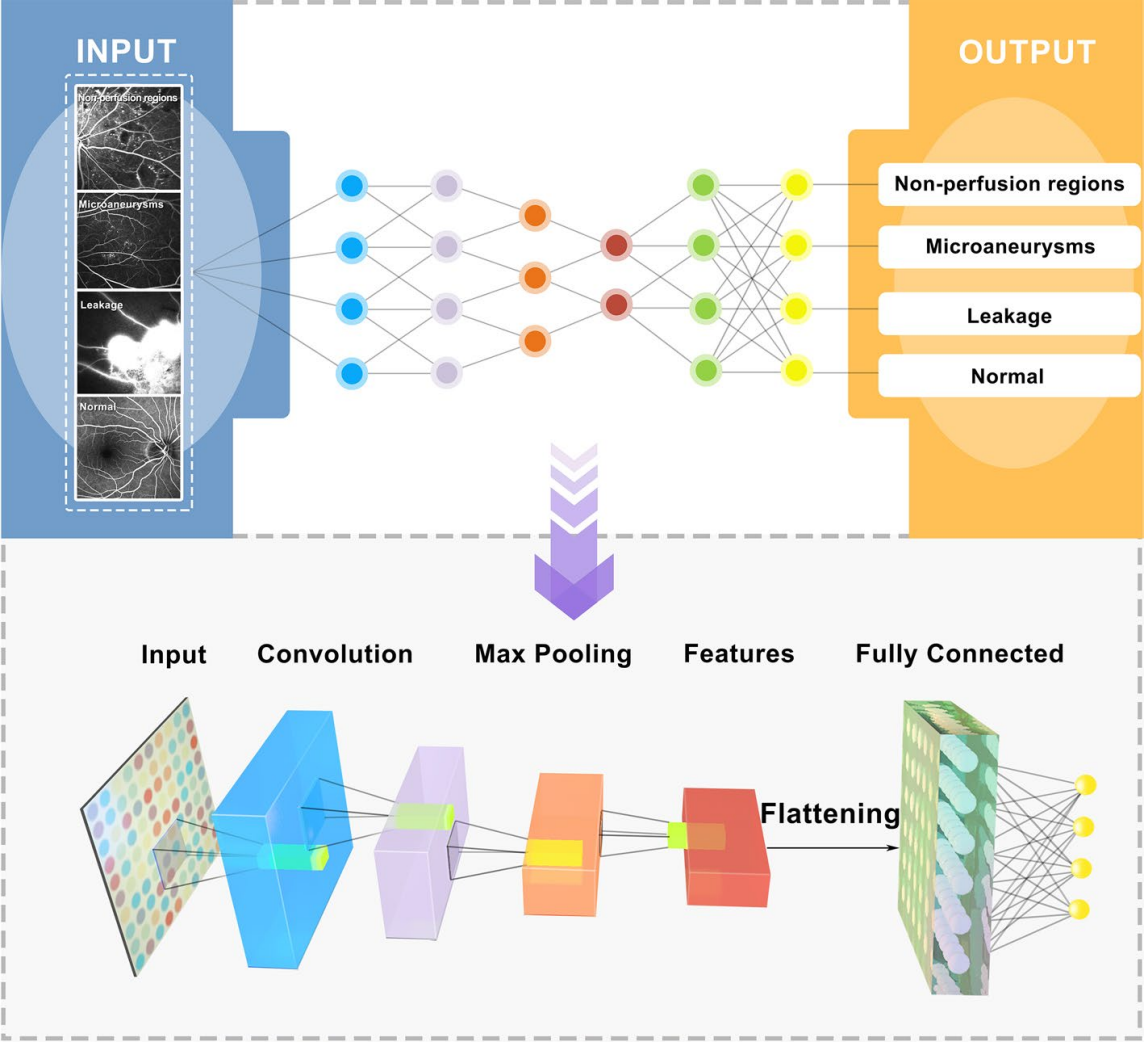
Abstraction of the proposed algorithmic pipeline for DR lesion classification.

The characteristics and distribution of the dataset is shown in Table 1. A CNN model for multi-label classification is constructed, which takes a single channel grayscale image as input and outputs a 4-length vector for 3 classes: (1) NP regions, (2) microaneurysms, (3) leakage. Each dimension in the output represents whether the lesion is contained in the input image, and if none of them exists, the image is normal. The architecture of the CNN is a DenseNet with 4 dense blocks, each of them has 16 dense layers with a grow rate of 12. An overall structure of this deep learning model is represented in Fig. 1. The CNN was trained with stochastic gradient descent (SGD) optimizer. The performance of the algorithm was evaluated by the area under the curve (AUC) of receiver operation characteristic curve (ROC) generated by plotting sensitivity (true positive rate) vs specificity (true negative rate).

### Automatic segmentation of NP regions

**Figure 2:**
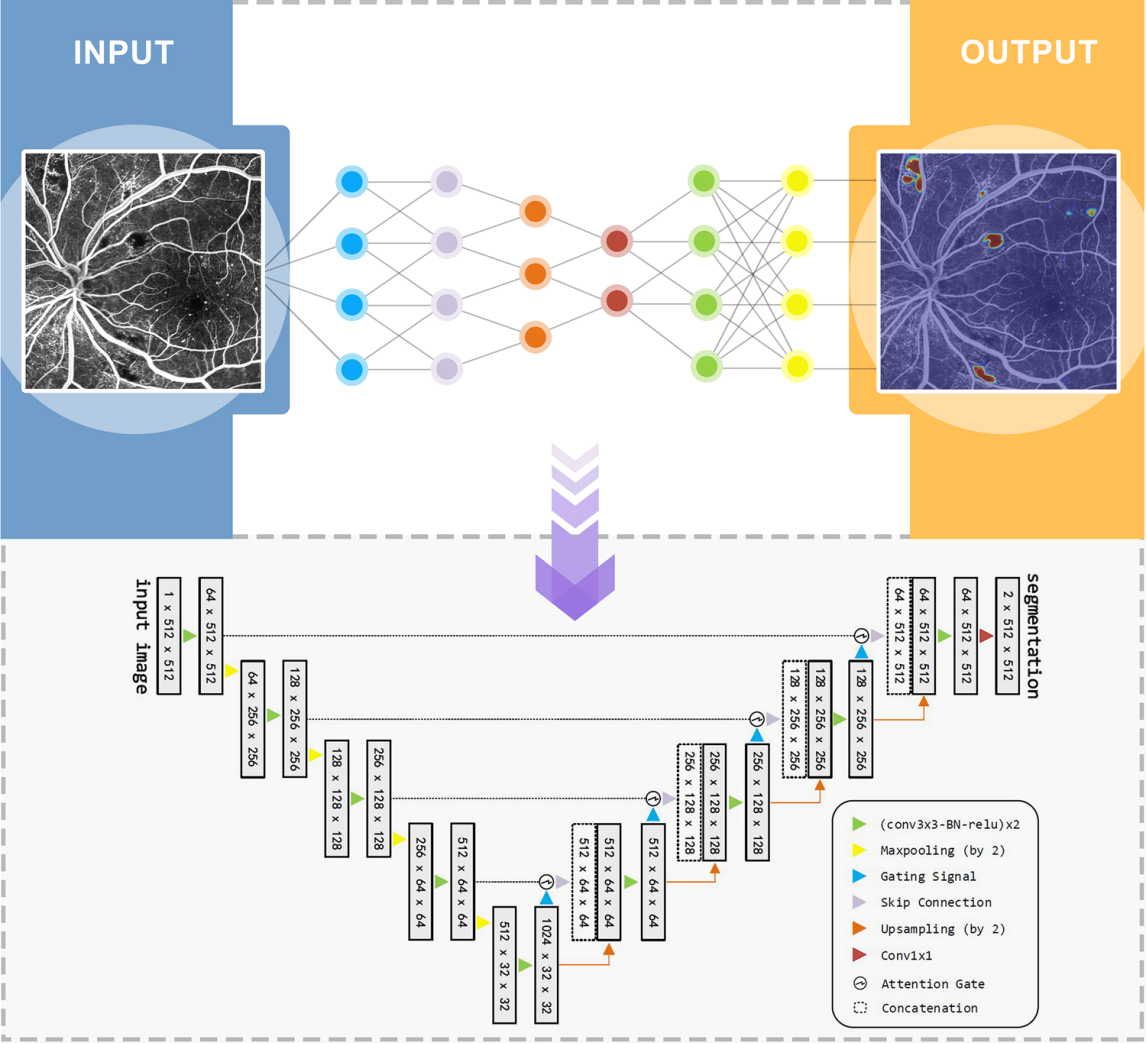
Structure of the U-net for automatic detection of non-perfusion (NP) regions.

We constructed an attention U-net for NP region detection with a down-sampling path and an up-sampling path, as shown in Fig. 2. In the down sampling path, 64*64-sized feature maps are generated by 4 max-pooling operations. Before each down-sampling, two composition blocks containing 3*3 convolution, batch normalization (BN) and rectified linear unit (ReLU) are adopted. The final feature maps of the down sampling path are up sampled to the original image size in 4 steps in the up-sampling path.

The NP dataset has 246 images, each of them contains NP regions. It was divided into training set and test set by a ratio of 5:1 (205:41). Data augmentation like that for FFA classification was applied. We computed the recall of proposals at different Intersection-over-Union (IoU) ratios with NP ground truth boxes.

## Results

**Figure 3:**
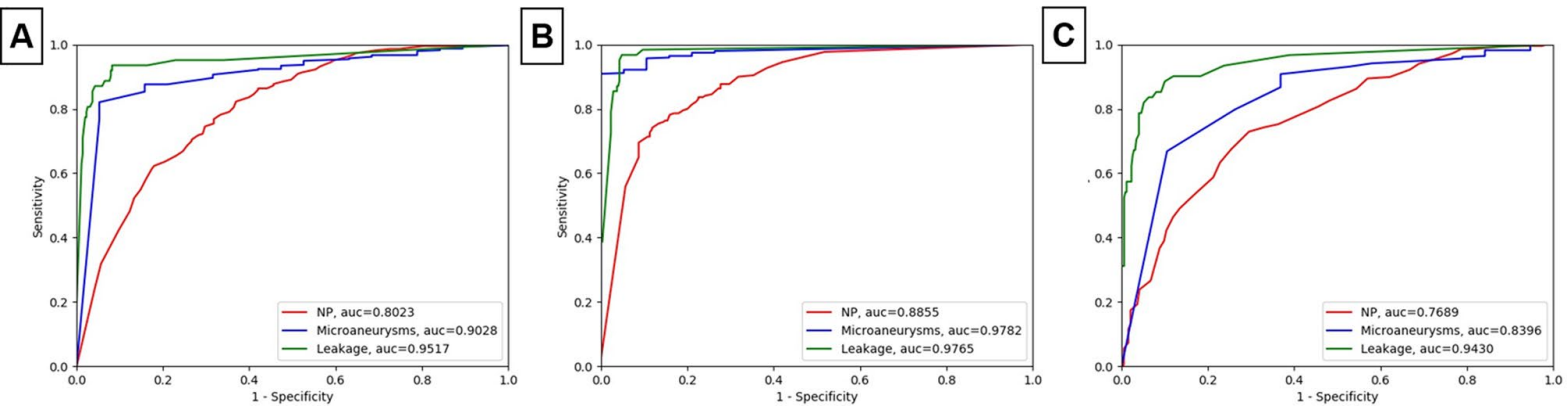
Verification of the performance of the three CNN models. (**A**) DenseNet, (**B**) ResNet 50, (**C**) VGG 16. Receiver operating characteristic (ROC) curves demonstrating the accuracy of lesions classification.

The FFA dataset has 3,014 images, of which 2,801 images contain microaneurysms, 1,565 contain NP regions, and 579 contain leakage. Results of prediction performance for different lesions on FFA images are shown in Fig. 3. For each DR lesion, we can use these plots to derive the sensitivity, specificity and AUC of three CNN models. To assess the statistical sensitivity we plotted each ROC curve 1,000 times. For DenseNet, AUC was 0.8855 for NP regions, 0.9782 for microaneurysms, and 0.9765 for leakage classifier. For ResNet 50, AUC was 0.7895 for NP regions, 08,633 for microaneurysms, and 0.9305 for leakage classifier. For VGG 16, AUC was 0.7689 for NP regions, 0.8396 for microaneurysms, and 0.9430 for leakage classifier. The sensitivity and specificity of 3 doctor’s diagnosis are also shown in the Table 2.

**Table 2:** Sensitivities and specificities of the proposed CNN and 3 ophthalmologists.

	NP		Microaneurysm		Leakage	
	Sensitivity (%)	Specificity (%)	Sensitivity (%)	Specificity (%)	Sensitivity (%)	Specificity (%)
Doctor 1	91.2	82.5	97.8	82.2	94.1	88.0
Doctor 2	92.4	90.7	97.6	93.0	89.3	94.2
Doctor 3	94.6	89.7	99.4	79.6	74.3	99.4
DenseNet	87.3	86.1	96.1	73.3	80.4	97.8
ResNet50	72.3	70.8	25.2	94.7	64.2	98.3
VGG 16	63.3	77.2	66.8	89.5	78.7	26.6

**Figure 4:**
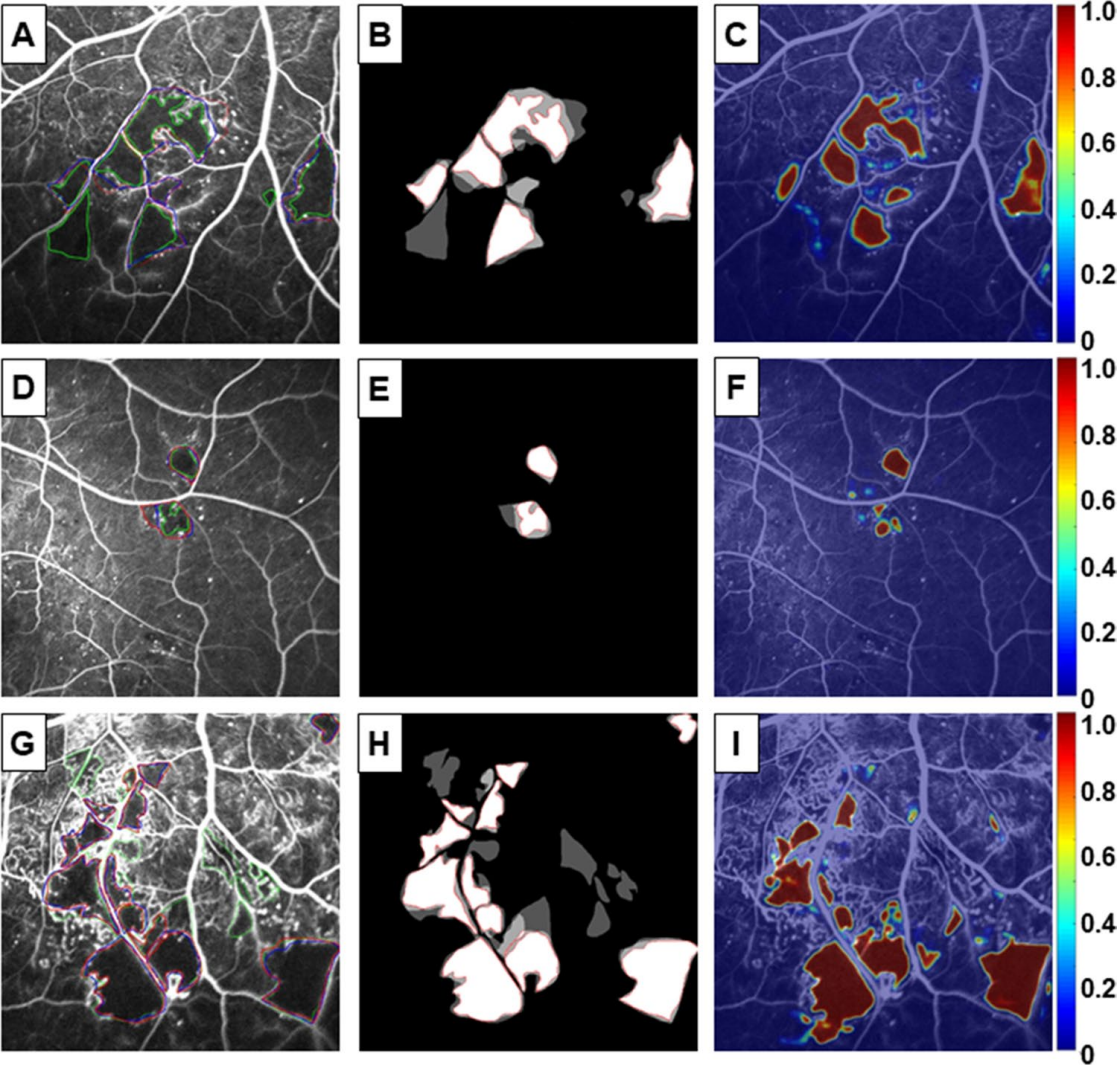
Prediction of NP regions using semantic segmentation. Manually segmentation of NP regions by three ophthalmologists is shown in the left column. The overlap areas of the results are illustrated in the center column. The AI algorithm provides a probability map for NP regions (right column).

**Figure 5:**
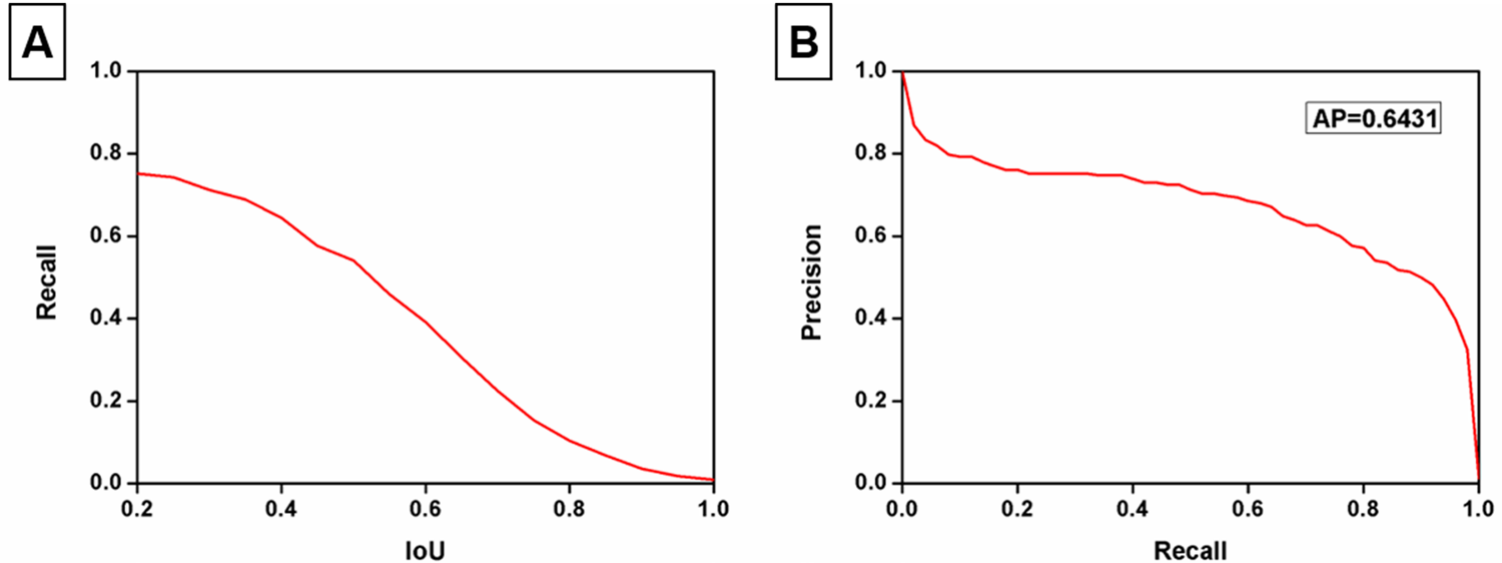
(**A**) Recall versus Intersection-over-Union (IoU) overlap ratio on the NP regions. (**B**) Precision-to-recall curve demonstrating the accuracies of NP detection. Average precision is 0.6431.

The outputs of NP regions are probability maps for each FFA image (Fig. 4). The first column pictures show the manually segmented ground-truth of three ophthalmologists by three different colors. The overlap areas of the ground-truth results are shown in the second column with white color. The third column shows the result of our algorithm’s prediction. Since the training data consisted of patches centered on the lesion of interest, the higher probability elevations in the probability map tended to cover both the lesion of interest and the surrounding pixels. In Fig. 5, we show the results of recall-to-IoU overlap ratio on the NP regions. Precision-to-recall curve demonstrates the accuracy of NP detection. Average precision is 0.643.

## Discussion

In this study, we have detected lesions of DME which need focal retinal photocoagulation. Excellent performance is reported in the detection of DR containing macroscopically visible lesions on the task of single-label image classification^9^. However, the applicability of CNNs to multi-label images still remains an open problem. In our study, DenseNet was first used to identify the multi-label lesions of microaneurysms, NP regions and leakage on FFA images. Multi-label classification using deep multi-model neural networks was reported with good result of whole slide breast histopathology images and X-ray images^15,16^. The use of many square image patches for training and detection with a sliding window technique has been employed in pathology of microaneurysm with success, but the limitation of MS-CNN is time efficiency^17^. We infer that DenseNet might be the appropriate CNN architecture for analysis of data modal of FFA images.

At the lesion level, the proposed detector outperforms heatmap generation algorithms of semantic segmentation based on FFA images. Classical lesion detection were segmentation methods based on mathematical morphology, region growing, or pixel classification, using fundus images^18–20^. Walid presented a framework for segmenting Choroidal neovascularization lesions based on parametric modeling of the intensity variation in FFA^21^. However, limited expert labeled data is a common problem encountered. CNNs based U-net structure was used to detect retinal exudates with promising results, and it does not rely on expert knowledge or manual segmentation for detecting relevant lesions^22^. Our proposed solution is a promising image mining tool, which has the potential to discover new biomarkers in images.

Precision treatment of DR lesions such as that prescribed by the ETDRS protocol requires the retina specialists locate each lesion seen on the FFA on the patient’s fundus, which is a tedious procedure with frequent retreatment sessions^23^. It is important to point out that most AI-based applications in medicine focus on the diagnosis or screening and few are involved with treatment. The NAVILAS system reported the first retinal navigating laser photocoagulation system in the treatment of DR, which is manually done offline with plentiful time^24^. The navigated laser in a randomized clinical trial resulted in significantly fewer injections and yielded visual and anatomic gains comparable to monthly dosing at 2 years^25^. In the clinical setting, we avoid the large vessels of the retina and shift the location of photocoagulation, when we perform the laser photocoagulation with the same spacing. We detect the defining lesions with an accuracy of 0.643 using computer-aided system, and plan the aiming laser spot on the retina.

There are limitations to this study. Annotating lesions on FFA images is costly, learning from the weakly supervised data helps to reduce the need for large-scale labeled training data in deep learning. Some NP Annotation task is also difficult for a human doctor as well. The FFA images and fundus images with same fields of view were manually registered, automatic registration will be done in the future. Our system helps clinical ophthalmologists make laser treatment decisions for diabetic retinopathy, the accuracy and safety should be evaluated by clinical trials in the future.

Although AI has got a series of achievements in ophthalmology, it still remains great challenges for the application in assisting doctors with the diagnosis and treatment of retinal diseases. Our algorithm can assist doctors reading the FFA images and guiding the laser treatment, maybe be used as an intelligent tool for FFA analysis in the future. Through this system, doctors can receive an AI report labelling with different lesions as soon as the test is finished, which will approve the doctors’ efficiency with the detection of abnormal findings that doctors may ignore. Moreover, the AI suggestion of laser treatment can also help junior doctors make decisions.
